# Psychiatric Diagnoses in Individuals with Non-Syndromic Oral Clefts: A Danish Population-Based Cohort Study

**DOI:** 10.1371/journal.pone.0156261

**Published:** 2016-05-25

**Authors:** Dorthe Almind Pedersen, George L. Wehby, Jeffrey C. Murray, Kaare Christensen

**Affiliations:** 1 Epidemiology, Biostatistics and Biodemography, Department of Public Health, University of Southern Denmark, Odense C, Denmark; 2 Department of Clinical Genetics, Department of Biochemistry and Pharmacology, Odense University Hospital, Odense C, Denmark; 3 Department of Health Management and Policy, University of Iowa College of Public Health, Iowa City, United States of America; 4 Department of Pediatrics, University of Iowa College of Medicine, Iowa City, United States of America; Johns Hopkins Bloomberg School of Public Health, UNITED STATES

## Abstract

**Background:**

The aim of this study was to investigate the risk of psychiatric diagnoses in individuals with non-syndromic oral clefts (OC) compared with individuals without OC, including ages from 1 to 76 years.

**Methods:**

Linking four Danish nationwide registers, we investigated the risk of psychiatric diagnoses at Danish psychiatric hospitals during the period 1969–2012 for individuals born with non-syndromic OC in Denmark 1936–2009 compared with a cohort of 10 individuals without OC per individual with OC, matched by sex and birth year. The sample included 8,568 individuals with OC, observed for 247,821 person-years, and 85,653 individuals without OC followed for 2,501,129 person-years.

**Results:**

A total of 953 (11.1%) of the individuals with OC (9.6% for cleft lip (CL), 10.8% for cleft lip and palate (CLP) and 13.1% for cleft palate (CP)) and 8,117 (9.5%) in the comparison group had at least one psychiatric diagnosis. Cox proportional hazard regression model revealed that individuals with OC had significantly higher risk of a psychiatric diagnosis (hazard ratio (HR) = 1.19, 95% CI: 1.12–1.28). When examining cleft type, no difference was found for CL (HR = 1.03, 95% CI: 0.90–1.17), but CLP was associated with a small increased risk (HR = 1.13, 95% CI: 1.01–1.26), whereas individuals with CP had the largest increased risk (HR = 1.45, 95% CI: 1.30–1.62). The largest differences were found in schizophrenia-like disorders, mental retardation and pervasive developmental disorders, but we found no increased risk of mood disorders and anxiety-related disorders.

**Conclusion:**

Individuals with non-syndromic OC had significantly higher risk of psychiatric diagnoses compared with individuals without OC. However, the elevated risk was observed for individuals with CLP and CP but not for individuals with CL and the absolute risk increase was modest.

## Introduction

Oral clefts (OC) including cleft lip only (CL), cleft lip with cleft palate (CLP) and cleft palate only (CP) are among the most common congenital malformations with a birth prevalence estimated at 1.4 per 1000 live births in Denmark [1, 2]. OC can be associated with other major anomalies or be part of a syndrome in which cases the OC is classified as syndromic. There is a strong genetic component to OC and cleft lip with or without cleft palate CL(P) has shown to be etiologically different from CP [3, 4].

OC can present several adverse consequences to the health and wellbeing of affected individuals throughout life. Depending on the type and severity of the cleft, affected children may experience feeding problems and often need surgical repair, long-term orthodontic care and speech therapy throughout childhood and into adolescence. Some studies have also suggested increased dissatisfaction with appearance and/or speech, peers’ teasing, lower social functioning, reduced academic achievement, increased utilization of healthcare services throughout most of the lifespan, and reduced quality of life [5–13]. Other studies have found no or minimal differences in overall psychosocial functioning of individuals with non-syndromic OC [14, 15]. Although there is a relatively large amount of research on the impact of OC on psychosocial wellbeing, the studies are often based on small sample sizes, and several lack a control group and robust measures of mental health conditions [16, 17].

There are at least three main channels that can potentially modify the risk of mental health problems among individuals with OC. First, the adverse consequences to health and psychosocial status throughout childhood and adolescence can increase this risk. Another potential link is through certain genetic pathways that may relate to both OC and mental health problems. While no such genes have been specifically identified, certain syndromic forms of OC, such as the 22q11 deletion syndrome (velocardiofacial/DiGeorges syndrome), which can include CP, has shown to be associated with a wide range of mental disorders, including schizophrenia-like psychotic disorders, mild retardation, autism and ADHD [18, 19]. Finally, several neuroimaging studies have reported abnormal brain structure in individuals with non-syndromic OC [20–23]. In particular, individuals with non-syndromic OC are found to have increased prevalence of midline brain anomaly [22, 23], which is reported to be associated with certain developmental disorders such as schizophrenia, mental retardation and developmental delay [24, 25].

The literature on psychiatric assessment of individuals with non-syndromic OC suggests that individuals with OC may experience specific problems such as increased rates of hyperactivity, impulsivity and inattention, depression and anxiety, elevated risk of suicide, and increased psychotropic drug use in adolescents [26–32]. However, much of the literature is based on self-reported measures which can be prone to bias as well as small and convenience samples which limit generalizability. Furthermore, most studies are focused on children and adolescents, leaving little known about mental health in this population throughout life.

This study investigates whether individuals with non-syndromic OC are at increased risk of severe mental disorders compared with individuals without OC using four nationwide registers from Denmark to assess psychiatric diagnoses at psychiatric hospitals over a period extending between 1969 and 2012. In addition to examining the risk of receiving care at a psychiatric hospital due to any psychiatric condition, we examine specific psychiatric diagnoses. Only one previous study has examined this question using large population-based data [33]. However, that study focused on psychiatric inpatient stays during 1969–1993. Moreover, it did not include a comparison cohort group, but was based on background population rates. The current study is an extension of this study which adds 19 years of follow-up and captures inpatient admissions and outpatient visits as well as emergency room visits for psychiatric problems, which gives a more sensitive instrument for identifying mental disorders. For the first time, the present study allows an examination of the mental health of individuals with OC throughout most of the lifespan, as it includes individuals up to age 76 years. Furthermore, our study includes birth cohorts from 1936 through 2009, which enables us to investigate psychiatric disorders specific to childhood and/or adolescence. This study is unprecedented in its sample size, representation, and objective measures, and it provides us with a unique opportunity to investigate specific psychiatric disorders not only for OC overall, but for specific cleft types.

## Material and Methods

### Data sources

This population-based cohort study was based on a linkage of the following four nationwide registers in Denmark:

*The Danish Facial Cleft Register* (DFCR) encompasses individuals born with OC in Denmark in the period 1936 to 2009. Registration and treatment of individuals with OC has been centralized in Denmark since the 1930s, which ensures high ascertainment for the complete cohort, and for CL(P) the ascertainment in the period 1983 to 1987 has been found to be 99% [34]. OC discovered later in life are also included. The register includes information on cleft type and severity of the cleft. Cleft lip (CL) includes cleft lip only. Cleft lip and cleft palate (CLP) includes cleft of the lip and cleft of the palate. Cleft palate (CP) includes cleft of the palate only and can be submucous, in the soft palate only or both the soft and the hard palate. Microforms such as bifid uvula, defects in the orbicularis oris muscle etc. are not included in the DFCR. Information on any associated anomalies are also registered and classified into major or minor depending on whether the anomaly is likely to be part of a syndrome. Major anomalies include neural tube defects, severe mental retardation, syndromes (e.g. Van der Woude, Pierre Robin, 22q11 deletion syndrome), whereas polydactyly, hip dislocation, clubfoot, umbilical hernia etc. are considered minor associated anomalies. Non-syndromic OC is defined in DFCR as individuals with no major associated anomalies or individuals with 0–2 minor associated anomalies. For the birth cohorts 1936–1987 the number of syndromic OC is likely underestimated [34], but medical records have been reviewed for the later birth cohorts in order to obtain more complete information on associated anomalies. In DFCR, 11% of CL(P) and 32% of CP are registered as syndromic. The register is described in details elsewhere [1, 2].

*The Danish Civil Registration System* (CRS) was established in 1968, where all persons alive and living in Denmark were registered and assigned a unique personal identification number. Since then all persons with permanent residence in Denmark are registered. This unique personal identification number enables unambiguous linkage of all national registers in Denmark. The CRS includes, among other variables, information on the unique personal identification number, sex, date of birth, birth registration code, date of death, and continuously updated information on date and place of residence [35]. The CRS was established for administrative purposes, and the information is generally accepted to be highly accurate [36].

*The Danish Psychiatric Central Research Register* (PCRR) comprises information on every inpatient admission to Danish psychiatric hospitals since 1969 and on all outpatient clinical visits and emergency room visits to these hospitals since 1995 [37]. The register contains information on dates of admission and discharge, start and end dates of outpatient treatment, dates of emergency visits and all diagnoses. Until December 1993, the 8th revision of the International Classification of Diseases (ICD-8) was used, and in 1994 and onwards the ICD-10 Classification of Mental and Behavioral Disorders: Diagnostic Criteria for Research (ICD-10-DCR) has been used. The PCRR includes only treatment in psychiatric hospital settings. Contacts with general practitioners or psychiatrists outside hospital settings where less severe psychiatric disorders may be treated are not included. Hospitalization due to severe psychiatric disorder takes place in psychiatric hospitals, and all psychiatric hospitals in Denmark are public and provide care free of charge. Therefore, the PCRR provides a complete nationwide assessment of all mental disorders treated in psychiatric hospitals, and several of the key diagnoses have been validated with good results [38–42].

The *Danish Register of Causes of Death* contains computerized records on causes of death for all deaths among Danish residents dying in Denmark since 1970 [43]. The register contains the underlying cause of death and any contributory causes of death classified according to ICD-8 until December 1993 and according to ICD-10 in 1994 and onwards. Until 2007 coding was performed by specially trained coders under the supervision of the medical staff of the National Board of Health based on the medical information on the death certificates. Since 2007, the medical doctor, who has verified the death and issued the death certificate, also classifies these causes and the death certificates are submitted by an electronic form to the National Board of Health. The data quality relies on the notification done by the individual physicians and the coding in the National Board of Health. No central validation of the classification is done.

This study was approved by the Danish Data Protection Agency (Project No. 2016-41-4534) and no informed consent was required, because the study is based on secondary, de-identified data.

### Study population

**Fig 1:**
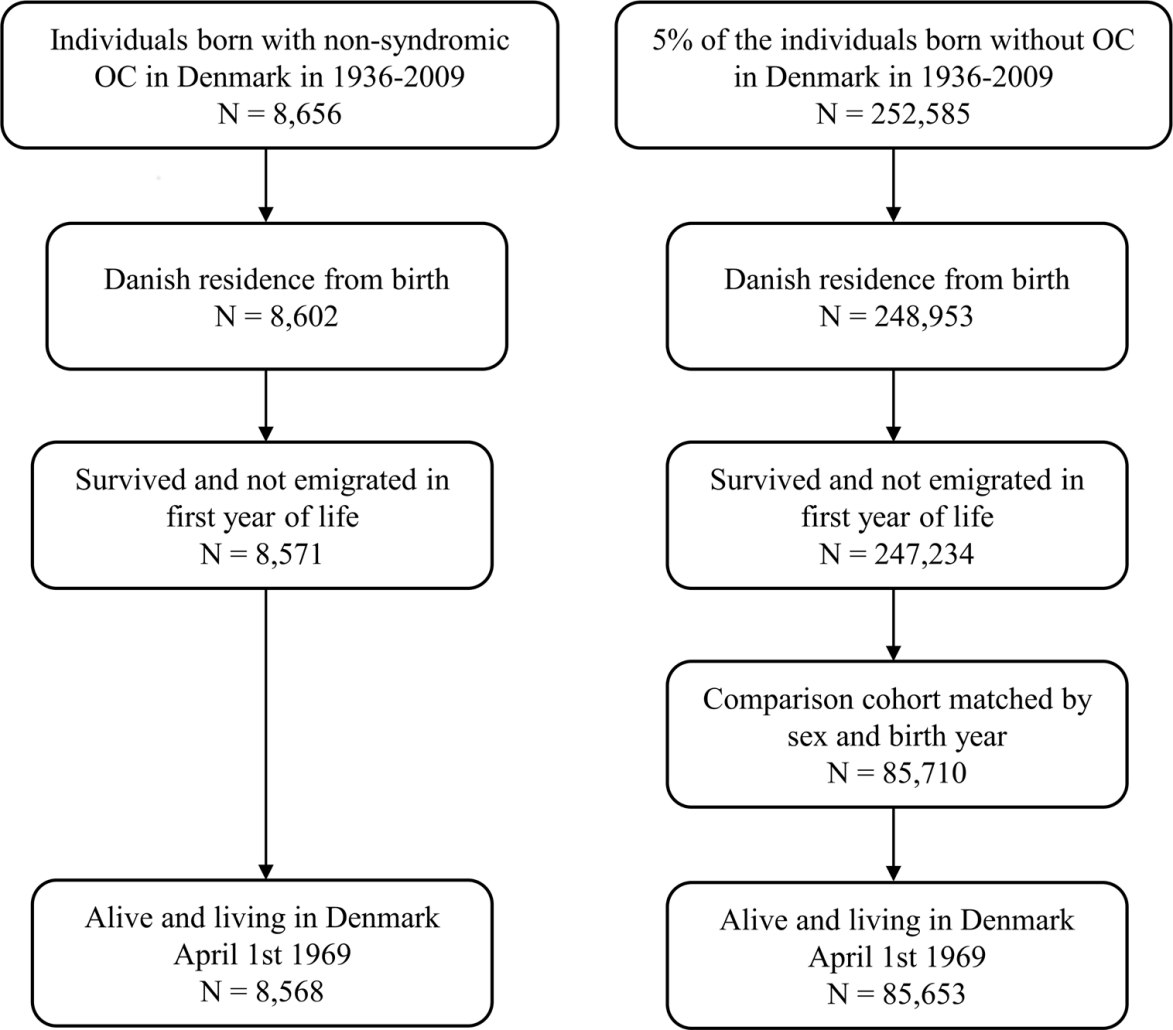
Inclusion Criteria for Individuals with Non-Syndromic Oral Cleft and Matched Comparison Cohort.

All individuals born January 1, 1936 to December 31, 2009 with non-syndromic OC were identified using DFCR. A 5% random sample of the entire population in Denmark in each year was extracted by Statistics Denmark. From this random sample we included only individuals without OC. In both groups we included only individuals with a Danish birth registration code and a Danish code of residence from birth, since the DFCR only includes individuals born in Denmark. Furthermore, in both groups we included only individuals who did not die or emigrate from Denmark in their first year of life. From the remaining 5% random sample, we randomly selected a comparison cohort of individuals without OC matched 10 to 1 to the group with OC by sex and birth year. Finally we restricted both groups to those alive and living in Denmark April 1, 1969 where registration of psychiatric admissions was initiated. The final study sample consisted of 8,568 individuals with OC and 85,653 in the comparison cohort. The ratio of number of individuals with OC to number of individuals in the comparison cohort is not precisely 1 to 10 in the final study sample, since individuals who died or emigrated prior to initiation of follow-up are excluded (Flow diagram is shown in Fig 1).

This study focuses on non-syndromic OC, but results for all individuals with OC and for individuals with syndromic OC can be seen in the supporting information (Flow diagram for all individuals with OC and for individuals with syndromic OC are shown in S1 and S2 Figs, respectively).

### Assessment of psychiatric disorder

We included all inpatient admissions, outpatient contacts and visits to emergency care units at a Danish psychiatric hospital initiated between April 1, 1969 and December 31, 2012 based on the PCRR. For ICD8 data, we excluded observations with a modification code, i.e. “suspected but not confirmed" diagnose, and for ICD-10-DCR data, we excluded referral diagnoses and temporal diagnoses since these are not necessarily confirmed. Individuals were classified with a psychiatric disorder if they received care at a psychiatric hospital with a psychiatric diagnosis (ICD-8:290–315 and ICD-10-DCR: F00-F99). The specific mental disorders were categorized according to Table 1 following the study [44], and the date of diagnosis of a specific mental disorder was set to be the first day of hospital encounter (inpatient, outpatient, or emergency care) indicating that disorder. Individuals with more than one diagnosis were included in each specific disorder, i.e. it is possible for individuals to be in more than one diagnosis group but obviously in the overall analyses of any psychiatric diagnosis we only included the first psychiatric diagnosis.

**Table 1:** Classification of Psychiatric Disorders According to ICD-10-DCR and equivalent ICD-8 Diagnoses.

Diagnosis	Earliest possible age at onset, y	ICD-10-DCR Codes	Equivalent ICD8-Codes
Any psychiatric disorder	1	F00-F99	290–315
Organic, including symptomatic, mental disorder (including dementia)	35	F00-F09	290.09, 290.10, 290.11, 290.18, 290.19, 292.x9, 293.x9, 294.x9, 309.x9
Mental and behavioral disorders due to psychoactive substance abuse	10	F10-F19	291.x9, 294.39, 303.x9, 303.20, 303.28, 303.90, 304.x9
Schizophrenia and related disorders (including schizophrenia, schizoaffective disorder)	10	F20-F29	295.x9, 296.89, 297.x9, 298.29–298.99, 299.04, 299.05, 299.09, 301.83
Mood disorders (including bipolar, single and recurrent depressive disorder)	10	F30-F39	296.x9 (excluding 296.89), 298.09, 298.19, 300.49, 301.19
Neurotic, stress-related, and somatoform disorders (including obsessive-compulsive disorder)	5	F40-F48	300.x9 (excluding 300.49) 305.x9, 305.68, 307.99
Eating disorders	1	F50	305.60, 306.50, 306.58, 306.59
Specific personality disorders (including borderline)	10	F60	301.x9 (excluding 301.19), 301.80, 301.81, 301.82, 301.84
Mental retardation (Intellectual disability)	1	F70-F79	311.xx, 312.xx, 313.xx, 314.xx, 315.xx
Pervasive developmental disorders (Autism spectrum disorder)	1	F84	299.00, 299.01, 299.02, 299.03
Behavioral and emotional disorders with onset usually occurring in childhood and adolescence (including hyperkinetic disorder)	1	F90-F98	306.x9, 308.0x

### Assessment of suicide

It has previously been shown using the DFCR that individuals with OC with and without associated anomalies have increased risk of suicide [30] and therefore we used the Danish Register of Causes of Death to identify individuals having suicide as underlying or contributory cause of death (ICD-8: E950-E959 and ICD-10: X60-X84 and Y87.0) from January 1, 1970 to December 31, 2010, in order to address the risk of suicide in individuals with non-syndromic OC.

### Study design and statistical analysis

Individuals were followed from April 1, 1969 or the earliest possible age at which the person could develop the specific mental disorder (see Table 1), whichever came later. Follow-up were terminated at date of first diagnosis of the specific disorder, death, first emigration from Denmark or December 31, 2012, whichever came first. Emigration from Denmark was defined as residence outside Denmark for more than 2 years.

Cox proportional hazard model with age as the underlying time scale was used to compute hazard ratios (HRs) of time to first diagnosis for individuals with OC compared with the matched comparison cohort for any psychiatric diagnosis and for each specific disorder. Additional models were stratified by cleft type and each cleft type was compared with their matched comparison cohort. Furthermore, models were estimated stratifying by birth cohort, sex and age. The proportional hazard assumption across age was assessed graphically.

## Results

A total of 8,568 individuals with non-syndromic OC were observed for 247,821 person-years and 85,653 individuals from the comparison cohort were followed for 2,501,129 person-years. Of the individuals with OC, 2,667 (31%) individuals had CL, 3,265 (38%) individuals had CLP, and 2,636 (31%) individuals had CP.

Table 2 summarizes the characteristics of individuals with OC and the matched comparison cohorts. The individuals with OC had significantly more deaths (Χ^2^ = 61.3, p < 0.001), including suicides (Χ^2^ = 4.6, p < 0.032), and fewer emigrations (Χ^2^ = 26.8, p < 0.001). A total of 953 (11.1%) of the individuals with OC (9.6% for CL, 10.8% for CLP and 13.1% for CP) and 8,117 (9.5%) in the comparison group received at least one diagnosis of a mental health disorder at a psychiatric hospital, which corresponds to a crude absolute risk increase of 0.3% (95% CI: -0.9%-1.4%) for CL, 1.1% (95% CI: 0.0%-2.2%) for CLP and 3.7% (95% CI: 2.4%-5.0%) for CP.

**Table 2:** Characteristics of Individuals with Non-Syndromic Oral Cleft and the Matched Comparison Cohort.

		Oral Cleft		Cleft lip		Cleft lip with cleft palate		Cleft palate	
		Affected individuals, n (%)	Comparison cohort, n (%)	Affected individuals, n (%)	Comparison cohort, n (%)	Affected individuals, n (%)	Comparison cohort, n (%)	Affected individuals, n (%)	Comparison cohort, n (%)
Total sample		8,568 (100.0)	85,653 (100.0)	2,667 (100.0)	26,668 (100.0)	3,265 (100.0)	32,643 (100.0)	2,636 (100.0)	26,342 (100.0)
Male		5,201 (60.7)	51,988 (60.7)	1,722 (64.6)	17,212 (64.5)	2,285 (70.0)	22,843 (70.0)	1,194 (45.3)	11,933 (45.3)
Year of birth									
	1936−1955	1,936 (22.6)	19,349 (22.6)	631 (23.7)	6,316 (23.7)	767 (23.5)	7,665 (23.5)	538 (20.4)	5,368 (20.4)
	1956−1975	2,742 (32.0)	27,404 (32.0)	842 (31.6)	8,412 (31.5)	1,054 (32.3)	10,538 (32.3)	846 (32.1)	8,454 (32.1)
	1976−1995	2,352 (27.5)	23,520 (27.5)	698 (26.2)	6,980 (26.2)	861 (26.4)	8,610 (26.4)	793 (30.1)	7,930 (30.1)
	1996−2009	1,538 (18.0)	15,380 (18.0)	496 (18.6)	4,960 (18.6)	583 (17.9)	5,830 (17.9)	459 (17.4)	4,590 (17.4)
Number of deaths^a^		648 (7.6)	4,717 (5.5)	189 (7.1)	1,499 (5.6)	269 (8.2)	1,969 (6.0)	190 (7.2)	1,249 (4.7)
Number of suicides in 1970–2010		40 (0.5)	279 (0.3)	12 (0.5)	97 (0.4)	21 (0.6)	116 (0.4)	7 (0.3)	66 (0.3)
Number of emigrations^a^		394 (4.6)	5,118 (6.0)	134 (5.0)	1,642 (6.2)	131 (4.0)	1,956 (6.0)	129 (4.9)	1,520 (5.8)
Number of persons alive and resident in Denmark at initiation of follow up									
	when earliest possible age of onset is 5 years	8,313 (97.0)	83,152 (97.1)	2,599 (97.5)	25,921 (97.2)	3,166 (97.0)	31,677 (97.0)	2,548 (96.7)	25,554 (97.0)
	when earliest possible age of onset is 10 years	7,811 (91.2)	78,051 (91.1)	2,446 (91.7)	24,375 (91.4)	2,970 (91.0)	29,666 (90.9)	2,395 (90.9)	24,010 (91.1)
	when earliest possible age of onset is 35 years	4,711 (55.0)	47,107 (55.0)	1,492 (55.9)	14,851 (55.7)	1,837 (56.3)	18,238 (55.9)	1,382 (52.4)	14,018 (53.2)
Any psychiatric disorder^a^		953 (11.1)	8,117 (9.5)	255 (9.6)	2,480 (9.3)	352 (10.8)	3,153 (9.7)	346 (13.1)	2,484 (9.4)
	Organic, including symptomatic, mental disorder^d^	43 (0.9)	328 (0.7)	15 (1.0)	109 (0.7)	16 (0.9)	122 (0.7)	12 (0.9)	97 (0.7)
	Mental and behavioral disorders due to psychoactive substance abuse^c^	248 (3.2)	2,102 (2.7)	60 (2.5)	660 (2.7)	108 (3.6)	869 (2.9)	80 (3.3)	573 (2.4)
	Schizophrenia and related disorders^c^	160 (2.0)	1,181 (1.5)	43 (1.8)	379 (1.6)	65 (2.2)	456 (1.5)	52 (2.2)	346 (1.4)
	Mood disorders^c^	251 (3.2)	2,366 (3.0)	67 (2.7)	674 (2.8)	91 (3.1)	910 (3.1)	93 (3.9)	782 (3.3)
	Neurotic, stress-related, and somatoform disorders^b^	363 (4.4)	3,538 (4.3)	92 (3.5)	1,062 (4.1)	129 (4.1)	1,316 (4.2)	142 (5.6)	1,160 (4.5)
	Eating disorders	37 (0.4)	301 (0.4)	15 (0.6)	80 (0.3)	8 (0.2)	103 (0.3)	14 (0.5)	118 (0.4)
	Specific personality disorders^c^	202 (2.6)	1,719 (2.2)	62 (2.5)	526 (2.2)	64 (2.2)	654 (2.2)	76 (3.2)	539 (2.2)
	Mental retardation	106 (1.2)	365 (0.4)	13 (0.5)	113 (0.4)	36 (1.1)	156 (0.5)	57 (2.2)	96 (0.4)
	Pervasive developmental disorders	67 (0.8)	409 (0.5)	14 (0.5)	126 (0.5)	19 (0.6)	184 (0.6)	34 (1.3)	99 (0.4)
	Behavioral and emotional disorders with onset usually occurring in childhood and adolescence	138 (1.6)	1,183 (1.4)	41 (1.5)	357 (1.3)	50 (1.5)	477 (1.5)	47 (1.8)	349 (1.3)

Percentages are of the total sample unless otherwise stated.

^a^ Numbers are the total number in the period April 1, 1969 to December 31, 2012.

^b^ Percentages are of number of persons alive and resident in Denmark at initiation of follow up when earliest possible age of onset is 5 years of age.

^c^ Percentages are of number of persons alive and resident in Denmark at initiation of follow up when earliest possible age of onset is 10 years of age.

^d^ Percentages are of number of persons alive and resident in Denmark at initiation of follow up when earliest possible age of onset is 35 years of age.

Table 3 shows that individuals with OC had a 19% (95% CI: 12%-28%) higher relative risk of having a diagnosis at a psychiatric hospital compared with the comparison cohort of individuals without OC. Stratified by cleft type, we found no significant difference from the unaffected group for individuals with CL (HR = 1.03, 95% CI: 0.90–1.17), a small but significantly increased risk for individuals with CLP (HR = 1.13, 95% CI: 1.01–1.26), and a larger increase in risk for individuals with CP (HR = 1.45, 95% CI: 1.30–1.62). Dividing into the specific types of psychiatric disorder, we found a significantly increased risk of mental and behavioral disorders due to psychoactive substance abuse, schizophrenia and related disorders, specific personality disorders, mental retardation, and pervasive developmental disorders for all OC. Furthermore, in individuals with CP we found a significantly increased risk of neurotic, stress-related and somatoform disorders, and behavioral and emotional disorders. For individuals with CP, HR-estimates were above one for all diagnosis groups. For individuals with CLP, HR-estimates for disorders due to psychoactive substance abuse, schizophrenia and related disorders and mental retardation were statistically significant. The only significant finding for individuals with CL was increased risk of eating disorder.

**Table 3:** Hazard Ratios of Contact to a Psychiatric Hospital for Specific Psychiatric Diagnoses among Individuals with Non-Syndromic Oral Cleft Compared With the Matched Comparison Cohort.

	Individuals with oral cleft		Individuals with cleft lip		Individuals with cleft lip and palate		Individuals with cleft palte	
	HR	95% CI	HR	95% CI	HR	95% CI	HR	95% CI
Any psychiatric disorder	1.19***	1.12–1.28	1.03	0.90–1.17	1.13*	1.01–1.26	1.45***	1.30–1.62
Organic, including symptomatic, mental disorder	1.33	0.96–1.83	1.46	0.85–2.51	1.25	0.73–2.13	1.28	0.70–2.34
Mental and behavioral disorders due to psychoactive substance abuse	1.20**	1.05–1.37	0.90	0.69–1.18	1.25*	1.03–1.54	1.46**	1.16–1.85
Schizophrenia and related disorders	1.37***	1.16–1.62	1.18	0.86–1.62	1.41*	1.08–1.84	1.54**	1.15–2.06
Mood disorders	1.07	0.94–1.23	1.00	0.78–1.29	1.00	0.81–1.25	1.22	0.98–1.51
Neurotic, stress-related, and somatoform disorders	1.04	0.93–1.15	0.85	0.68–1.05	0.99	0.83–1.19	1.26**	1.06–1.51
Eating disorders	1.22	0.86–1.72	1.86*	1.07–3.23	0.81	0.39–1.66	1.13	0.63–2.00
Specific personality disorders	1.18*	1.02–1.37	1.14	0.87–1.49	0.99	0.77–1.29	1.46**	1.15–1.86
Mental retardation	2.88***	2.32–3.59	1.16	0.65–2.06	2.34***	1.62–3.36	5.81***	4.17–8.10
Pervasive developmental disorders	1.63***	1.25–2.11	1.13	0.65–1.96	1.03	0.64–1.66	3.36***	2.27–4.98
Behavioral and emotional disorders with onset usually occurring in childhood and adolescence	1.17	0.98–1.40	1.15	0.83–1.58	1.05	0.78–1.40	1.38*	1.02–1.87

*p<0.05

**p<0.01

***p<0.001

When analyses were repeated for all OC including individuals with syndromic OC, we saw similar results (see S1 and S2 Tables). The overall HR-estimate for having any psychiatric diagnosis for all OC was slightly elevated (HR = 1.25, 95% CI: 1.17–1.33), which was primarily due to increased point estimates for mental retardation, pervasive developmental disorders, and behavioral and emotional disorders. We saw no increase in point estimates for disorders due to psychoactive substance abuse or schizophrenia and related disorders (results from analyses only including the individuals with syndromic OC are shown in S3 and S4 Tables).

Stratifying by birth cohorts (1936–1968 and 1969–2009), sex, and age groups (0–34 years and 35–76 years), respectively, revealed similar point estimates overall to the total sample (results not shown). A few exceptions are worth mentioning, though. The earlier born cohorts had significantly higher risk for mental retardation (compared to individuals without OC) than later born cohorts; furthermore, individuals with CLP in the latter cohort (born in 1969–2009) did not have a significantly increased risk of mental retardation. When stratifying by sex, the increase for behavioral and emotional disorders was only significant for males. In analyses by age groups, the point estimates for any psychiatric disorder were smaller and statistically insignificant in the older age group although schizophrenia and related disorders, mental retardation, and pervasive developmental disorders were statistically significant and larger in magnitude than those from the total sample.

Proportional hazard assumption was verified graphically and could not be rejected except for mental retardation and pervasive developmental disorders, for which the HRs were significantly larger in adolescence and adulthood than in childhood.

## Discussion

In this large, population-based cohort study, we found a significantly increased risk of being diagnosed with psychiatric disorders at a Danish psychiatric hospital for individuals with non-syndromic OC compared with individuals without OC. This overall increased risk was mainly driven by a larger risk for individuals with CP and a smaller but statistically significant increased risk for CLP. However, individuals with CL did not show an elevated risk. The increased risks for several disorders associated with CP and CLP continue into older ages (35–76 years), especially schizophrenia and related disorders, mental retardation, and pervasive developmental disorders. While we focus on non-syndromic OC, the results are generally similar when adding syndromic cases (S2 Table) and we find similar point estimates overall and for the same specific disorders as in our previous study [33].

Our finding of higher risk of psychiatric disorders for individuals with CP is consistent with a recent Swedish register-based study which reported greater use of psychotropic medication among adolescents with CP compared to unaffected peers [31]. That study, however, also found increased use for individuals with CL, but not for CLP, a result opposite to our finding. This difference in findings for CL may arise because we mainly investigate the more severe psychiatric disorders, whereas the Swedish study also captures less severe disorders that are mainly treated in primary care. We do, however, find an increased risk of eating disorder for individuals with CL, which might support their finding. Their finding of no effect for individuals with CLP might be due to combining all types of psychotropic medication which may hide differences in the use of drugs for specific disorders.

Our finding of a higher risk of schizophrenia-related disorders, mental retardation and pervasive developmental disorder among individuals with non-syndromic OC are in line with reports of an association between non-syndromic OC and midline brain anomalies [22, 23]; these brain differences have been associated with schizophrenia, mental retardation and developmental delay in other studies [24, 25]. Furthermore, the increased risk of schizophrenia and related disorders supports previous reports of increased frequency of CP among patients with schizophrenia [45]. Our finding of a higher risk of behavioral and emotional disorders only for males is consistent with previous studies reporting increased rates of hyperactivity, impulsivity and inattention for males with non-syndromic OC [28, 29]. The finding of no significant differences for mood disorders or anxiety-related disorders is somewhat inconsistent with studies reporting increased rates of depression and social anxiety [26] and separation anxiety in children with OC [27]. This might again be because we investigate the more severe, mental disorders treated in hospital settings, in contrast to studies with a greater representation of milder psychiatric disorders like mild to moderate depression and anxiety.

Overall, our finding of increased risks of schizophrenia-like disorders, mental retardation and autism and not for mood disorders and anxiety-related disorders suggests that neurological differences or common genetic disposition may be more likely the underlying mechanisms for the association between OC and psychiatric diseases rather than psychological consequences due to having OC.

### Strengths and limitations

The major strengths of this study are the large sample size and long follow-up time which includes ages from 1 to 76 years. There is almost no selection bias as the project is register-based and information on OC and psychiatric diagnoses is collected independently. As previously mentioned, the ascertainment of individuals with OC in the population is virtually complete [34], and several of the psychiatric diagnoses are validated with good results [38–42].

Our results might be biased if individuals with OC are more likely to be referred to a psychiatric hospital, because of their greater use of healthcare services in general. This concern mainly applies to estimates for children with OC rather than adults since differences in hospitalizations and healthcare use compared to unaffected individuals are largest during childhood [9]. This would lead to overestimated relative risks for children. However, we find smaller risks for mental retardation and pervasive developmental disorders during childhood than in adolescence/adulthood, indicating that this is unlikely to be a major bias.

Another concern is differential loss to follow-up since individuals with non-syndromic OC have significantly more deaths and fewer emigrations during the follow-up period than individuals in the comparison cohort, which could potentially underestimate the association between OC and psychiatric diagnoses. In particular, the increased risk of suicide among individuals with OC would underestimate the association, but approximately half of those committing suicide receive a psychiatric diagnosis and the number of suicides is low compared to the number of individuals with a psychiatric disorder, so this is probably not affecting the estimates notably.

Furthermore, while we are likely to capture initial psychiatric diagnoses and incidence of disorders for most of the later-born cohorts, we may not capture all incidences for earlier-born cohorts and instead measure prevalence. However, this is likely to affect individuals with OC and the unaffected cohort in the same way and should thus not bias the results. It mainly involves the early birth cohorts and thereby the older age group, in which cases the increased relative risks may not be due to newly diagnosed psychiatric disorders, but could be driven by more re-admissions of prevalent cases.

Finally, it is possible that some individuals classified as non-syndromic OC in our analyses have unidentified associated anomalies that can also relate to increased risks of mental disorders. This is likely to be more of a concern for earlier birth cohorts that have greater underreporting of associated anomalies, but should be less of a concern for our estimates specific to later-born cohorts (S3 Fig illustrates the proportion of associated anomalies for individuals with OC by birth decades). Furthermore, analyses adding all syndromic and non-syndromic cases together did not substantially increase the risk estimates, indicating that any missed syndromic cases are unlikely to inflate the results for the non-syndromic group to any substantial degree. Furthermore, we expect that the major anomalies are most likely detected and, therefore, primarily the minor anomalies will be undetected; however, we cannot rule out the possibility that some of the increased risk is due to undetected syndromes among individuals with OC, such as 22q11 deletion syndrome. One limitation of register-based studies such as ours is the inability to contact individuals to conduct more detailed phenotyping and genotyping.

### Conclusions

Individuals with non-syndromic OC in Denmark have a higher risk of being diagnosed with a psychiatric disorder at a psychiatric hospital compared to individuals without OC. However, the absolute risk increase is modest and the largest risk is for individuals with CP followed by CLP, whereas the risk is not elevated for individuals with CL. These increased risks are observed at both younger and older ages. Risks are mainly higher for schizophrenia-like disorders and mental retardation for individuals with CLP or CP and for personality disorders and pervasive developmental disorder for those with CP. There is no increased risk of mood disorders and anxiety-related disorders, however, which suggests that neurological differences or common genetic disposition are more plausible mechanisms for the association between OC and psychiatric disorders rather than the psychological consequences of being born with OC.

## Supporting Information

S1 Fig Inclusion Criteria for All Individuals with Oral Cleft and the Matched Comparison Cohort. (TIF)

S2 Fig Inclusion Criteria for Individuals with Syndromic Oral Cleft and the Matched Comparison Cohort. (TIF)

S3 Fig Proportion of Associated Anomalies by Birth Cohorts for each Cleft Type. (TIF)

S1 Table Characteristics of All Individuals with Oral Cleft and the Matched Comparison Cohort. (DOCX)

S2 Table Hazard Ratios of Contact to a Psychiatric Hospital for Specific Psychiatric Disorders among All Individuals with Oral Cleft Compared with the Matched Comparison Cohort. (DOCX)

S3 Table Characteristics of Individuals with Syndromic Oral Cleft and the Matched Comparison Cohort. (DOCX)

S4 Table Hazard Ratios of Contact to a Psychiatric Hospital for Specific Psychiatric Disorders among Individuals with Syndromic Oral Cleft Compared with the Matched Comparison Cohort. (DOCX)
