# Supplementary figures and images for: Psychiatric Diagnoses in Individuals with Non-Syndromic Oral Clefts: A Danish Population-Based Cohort Study
Source: PLoS One. 2016 May 25;11(5):e0156261. doi: 10.1371/journal.pone.0156261 (PMC4880322; doi:10.1371/journal.pone.0156261)

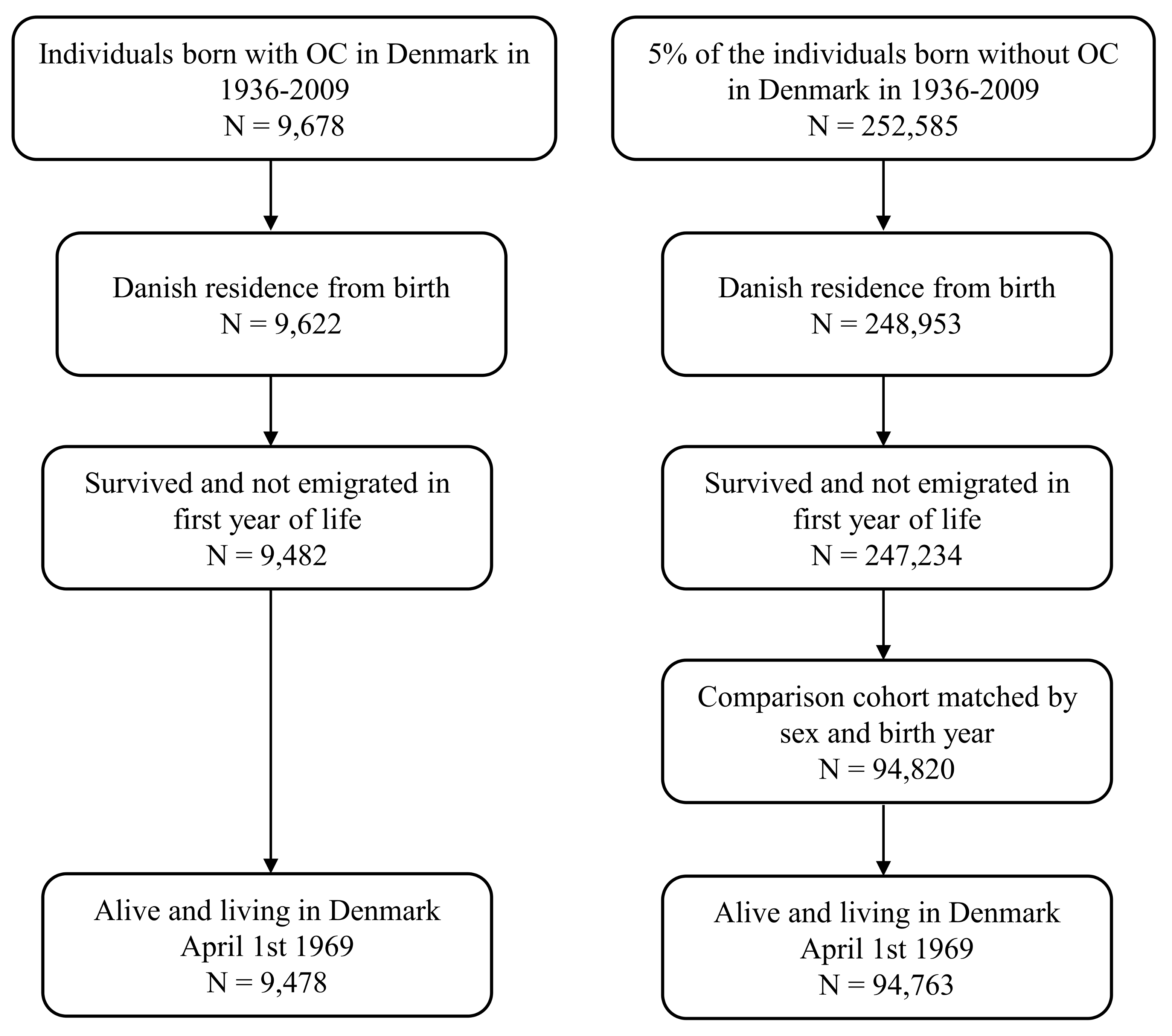

Supplement: S1 Fig — (TIF) [file pone.0156261.s001.tif]

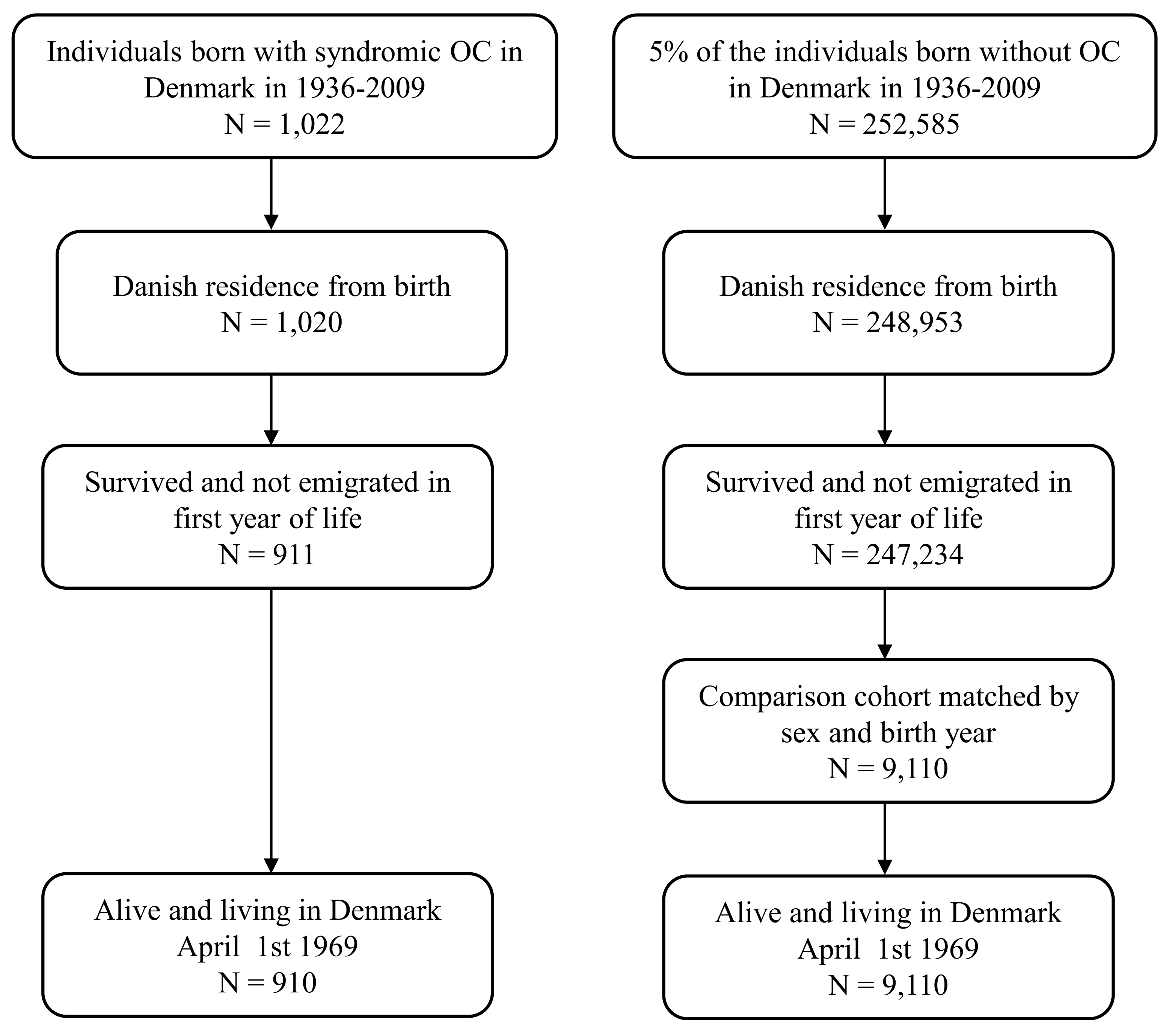

Supplement: S2 Fig — (TIF) [file pone.0156261.s002.tif]

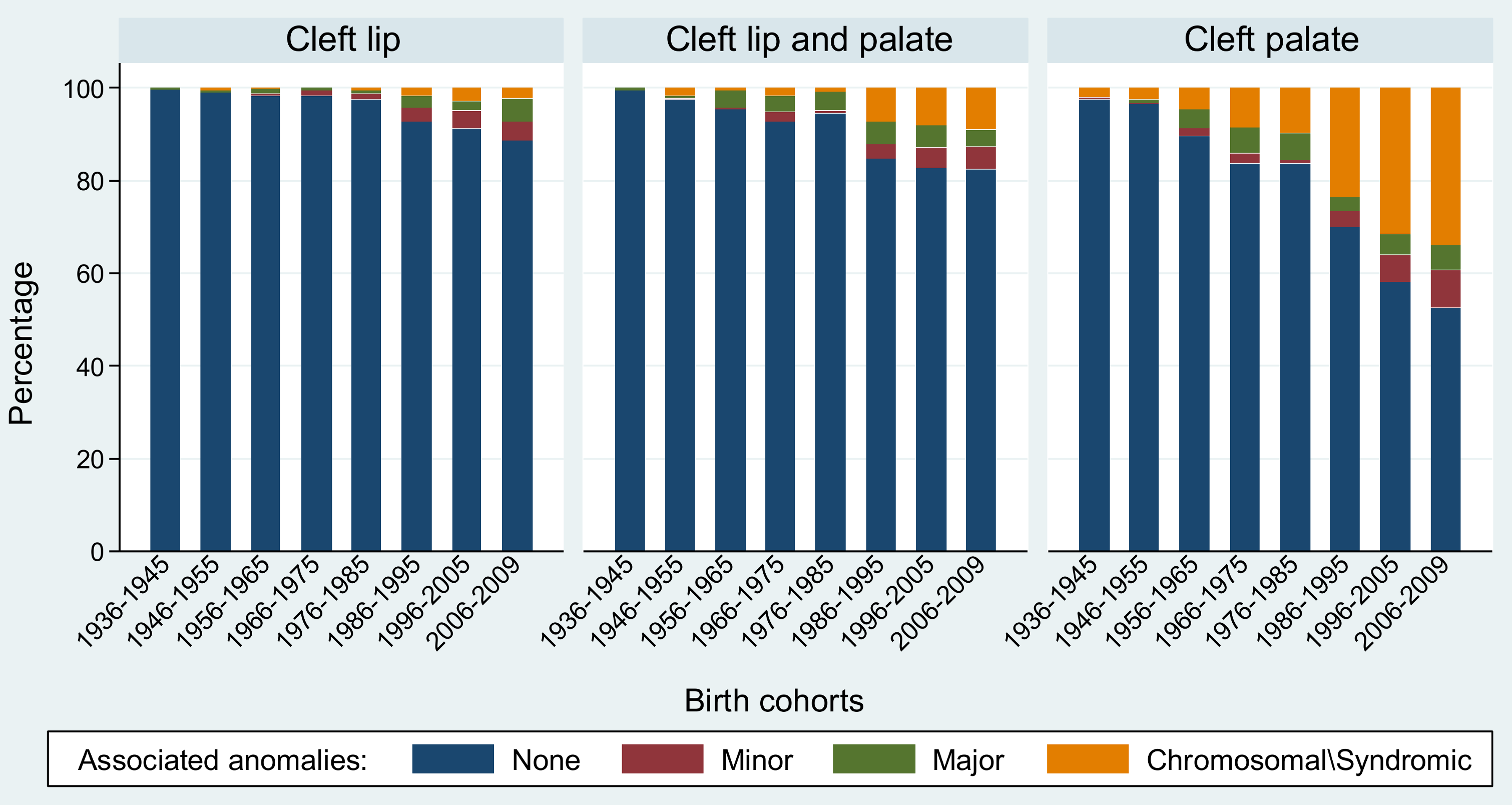

Supplement: S3 Fig — (TIF) [file pone.0156261.s003.tif]
