# Supplementary material for: Psychiatric Diagnoses in Individuals with Non-Syndromic Oral Clefts: A Danish Population-Based Cohort Study
Source: PLoS One. 2016 May 25;11(5):e0156261. doi: 10.1371/journal.pone.0156261 (PMC4880322; doi:10.1371/journal.pone.0156261)
Supplement: S1 Table — (DOCX) [file pone.0156261.s004.docx]

|  |  | Oral cleft | | Cleft lip | | Cleft lip and palate | | Cleft palate | |
| --- | --- | --- | --- | --- | --- | --- | --- | --- | --- |
|  |  | Affected individuals, n (%) | Comparison cohort, n (%) | Affected individuals, n (%) | Comparison cohort, n (%) | Affected individuals, n (%) | Comparison cohort, n (%) | Affected individuals, n (%) | Comparison cohort, n (%) |
| Total sample | | 9,478 (100.0) | 94,763 (100.0) | 2,729 (100.0) | 27,288 (100.0) | 3,501 (100.0) | 35,003 (100.0) | 3,248 (100.0) | 32,472 (100.0) |
| Male | | 5,690 (60.0) | 56,878 (60.0) | 1,761 (64.5) | 17,602 (64.5) | 2,437 (69.6) | 24,363 (69.6) | 1,492 (45.9) | 14,913 (45.9) |
| Year of birth | |  |  |  |  |  |  |  |  |
|  | 1936−1955 | 1,968 (20.8) | 19,669 (20.8) | 636 (23.3) | 6,366 (23.3) | 779 (22.3) | 7,785 (22.2) | 553 (17.0) | 5,518 (17.0) |
|  | 1956−1975 | 2,914 (30.7) | 29,134 (30.7) | 850 (31.1) | 8,492 (31.1) | 1,106 (31.6) | 11,058 (31.6) | 958 (29.5) | 9,584 (29.5) |
|  | 1976−1995 | 2,673 (28.2) | 26,730 (28.2) | 718 (26.3) | 7,180 (26.3) | 946 (27.0) | 9,460 (27.0) | 1,009 (31.1) | 10,090 (31.1) |
|  | 1996−2009 | 1,923 (20.3) | 19,230 (20.3) | 525 (19.2) | 5,250 (19.2) | 670 (19.1) | 6,700 (19.1) | 728 (22.4) | 7,280 (22.4) |
| Number of deaths^a^ | | 738 (7.8) | 4,852 (5.1) | 195 (7.1) | 1,513 (5.5) | 307 (8.8) | 2,022 (5.8) | 236 (7.3) | 1,317 (4.1) |
| Number of suicides in 1970-2010 | | 42 (0.4) | 289 (0.3) | 12 (0.4) | 98 (0.4) | 22 (0.6) | 119 (0.3) | 8 (0.3) | 72 (0.2) |
| Number with any emigration^a^ | | 414 (4.4) | 5,510 (5.8) | 136 (5.0) | 1,667 (6.1) | 135 (3.9) | 2,063 (5.9) | 143 (4.4) | 1,780 (5.5) |
| Number of persons alive and resident in Denmark at initiation of follow up | |  |  |  |  |  |  |  |  |
|  | when earliest possible age of onset is 5 years | 9,141 (96.4) | 91,703 (96.8) | 2,653 (97.2) | 26,473 (97.0) | 3,373 (96.3) | 33,903 (96.9) | 3,115 (95.9) | 31,327 (96.5) |
|  | when earliest possible age of onset is 10 years | 8,482 (89.5) | 85,149 (89.9) | 2,487 (91.1) | 24,817 (90.9) | 3,143 (89.8) | 31,560 (90.2) | 2,852 (87.8) | 28,772 (88.6) |
|  | when earliest possible age of onset is 35 years | 4,891 (51.6) | 49,246 (52.0) | 1,504 (55.1) | 14,986 (54.9) | 1,890 (54.0) | 18,914 (54.0) | 1,497 (46.1) | 15,346 (47.3) |
| Any psychiatric disorder^a^ | | 1,082 (11.4) | 8,862 (9.4) | 262 (9.6) | 2,522 (9.2) | 387 (11.1) | 3,352 (9.6) | 433 (13.3) | 2,988 (9.2) |
|  | Organic, including symptomatic, mental disorder^d^ | 44 (0.9) | 338 (0.7) | 16 (1.1) | 110 (0.7) | 16 (0.8) | 125 (0.7) | 12 (0.8) | 103 (0.7) |
|  | Mental and behavioral disorders due to psychoactive substance abuse^c^ | 260 (3.1) | 2,230 (2.6) | 60 (2.4) | 670 (2.7) | 112 (3.6) | 901 (2.9) | 88 (3.1) | 659 (2.3) |
|  | Schizophrenia and related disorders^c^ | 167 (2.0) | 1,258 (1.5) | 43 (1.7) | 386 (1.6) | 66 (2.1) | 473 (1.5) | 58 (2.0) | 399 (1.4) |
|  | Mood disorders^c^ | 271 (3.2) | 2,538 (3.0) | 69 (2.8) | 679 (2.7) | 96 (3.1) | 956 (3.0) | 106 (3.7) | 903 (3.1) |
|  | Neurotic, stress-related, and somatoform disorders^b^ | 395 (4.3) | 3,858 (4.2) | 94 (3.5) | 1,075 (4.1) | 135 (4.0) | 1,395 (4.1) | 166 (5.3) | 1,388 (4.4) |
|  | Eating disorders | 39 (0.4) | 339 (0.4) | 15 (0.5) | 81 (0.3) | 9 (0.3) | 116 (0.3) | 15 (0.5) | 142 (0.4) |
|  | Specific personality disorders^c^ | 211 (2.5) | 1,835 (2.2) | 63 (2.5) | 532 (2.1) | 66 (2.1) | 681 (2.2) | 82 (2.9) | 622 (2.2) |
|  | Mental retardation | 158 (1.7) | 408 (0.4) | 14 (0.5) | 116 (0.4) | 50 (1.4) | 164 (0.5) | 94 (2.9) | 128 (0.4) |
|  | Pervasive developmental disorders | 103 (1.1) | 493 (0.5) | 16 (0.6) | 131 (0.5) | 29 (0.8) | 209 (0.6) | 58 (1.8) | 153 (0.5) |
|  | Behavioral and emotional disorders with onset usually occurring in childhood and adolescence | 177 (1.9) | 1,380 (1.5) | 43 (1.6) | 373 (1.4) | 66 (1.9) | 538 (1.5) | 68 (2.1) | 469 (1.4) |
| Percentages is of the total sample unless otherwise stated | |  |  |  |  |  |  |  |  |
| ^a^ Numbers are the total number in the period April 1, 1969 to December 31, 2012. | | | | | | |  |  |  |
| ^b^ Percentages is of number of persons alive and resident in Denmark at initiation of follow up when earliest possible age of onset is 5 years of age. | | | | | | |  |  |  |
| ^c^ Percentages is of number of persons alive and resident in Denmark at initiation of follow up when earliest possible age of onset is 10 years of age. | | | | | | |  |  |  |
| ^d^ Percentages is of number of persons alive and resident in Denmark at initiation of follow up when earliest possible age of onset is 35 years of age. | | | | | | |  |  |  |
