# Supplementary material for: Psychiatric Diagnoses in Individuals with Non-Syndromic Oral Clefts: A Danish Population-Based Cohort Study
Source: PLoS One. 2016 May 25;11(5):e0156261. doi: 10.1371/journal.pone.0156261 (PMC4880322; doi:10.1371/journal.pone.0156261)
Supplement: S2 Table — (DOCX) [file pone.0156261.s005.docx]

|  |  | Individuals with oral cleft | |  | Individuals with cleft lip | |  | Individuals with cleft lip and palate | |  | Individuals with cleft palte | |
| --- | --- | --- | --- | --- | --- | --- | --- | --- | --- | --- | --- | --- |
|  |  | HR | 95% CI |  | HR | 95% CI |  | HR | 95% CI |  | HR | 95% CI |
| Any psychiatric disorder |  | 1.25*** | 1.17-1.33 |  | 1.04 | 0.92-1.18 |  | 1.17** | 1.05-1.30 |  | 1.52*** | 1.37-1.68 |
| Organic, including symptomatic, mental disorder |  | 1.32 | 0.96-1.82 |  | 1.55 | 0.91-2.62 |  | 1.22 | 0.72-2.09 |  | 1.22 | 0.67-2.21 |
| Mental and behavioral disorders due to psychoactive substance abuse |  | 1.19** | 1.05-1.35 |  | 0.89 | 0.68-1.16 |  | 1.25** | 1.03-1.53 |  | 1.42** | 1.13-1.77 |
| Schizophrenia and related disorders |  | 1.35*** | 1.14-1.59 |  | 1.16 | 0.85-1.59 |  | 1.37** | 1.05-1.78 |  | 1.50** | 1.14-1.98 |
| Mood disorders |  | 1.09 | 0.96-1.23 |  | 1.03 | 0.80-1.32 |  | 1.01 | 0.81-1.24 |  | 1.22 | 0.99-1.49 |
| Neurotic, stress-related, and somatoform disorders |  | 1.04 | 0.93-1.15 |  | 0.86 | 0.69-1.06 |  | 0.99 | 0.83-1.18 |  | 1.23** | 1.05-1.45 |
| Eating disorders |  | 1.15 | 0.82-1.61 |  | 1.84* | 1.06-3.20 |  | 0.81 | 0.41-1.60 |  | 1.02 | 0.59-1.76 |
| Specific personality disorders |  | 1.16* | 1.00-1.34 |  | 1.14 | 0.88-1.50 |  | 0.99 | 0.77-1.28 |  | 1.36** | 1.08-1.72 |
| Mental retardation |  | 3.87*** | 3.22-4.66 |  | 1.22 | 0.70-2.12 |  | 3.05*** | 2.21-4.20 |  | 7.37*** | 5.64-9.63 |
| Pervasive developmental disorders |  | 2.10*** | 1.70-2.60 |  | 1.24 | 0.74-2.09 |  | 1.40 | 0.95-2.06 |  | 3.79*** | 2.80-5.14 |
| Behavioral and emotional disorders with onset usually occurring in childhood and adolescence |  | 1.30** | 1.11-1.52 |  | 1.15 | 0.84-1.58 |  | 1.24 | 0.96-1.60 |  | 1.49** | 1.16-1.93 |
| * p<0.05, **p<0.01, ***p<0.001 |  |  |  |  |  |  |  |  |  |  |  |  |
