# Supplementary material for: Psychiatric Diagnoses in Individuals with Non-Syndromic Oral Clefts: A Danish Population-Based Cohort Study
Source: PLoS One. 2016 May 25;11(5):e0156261. doi: 10.1371/journal.pone.0156261 (PMC4880322; doi:10.1371/journal.pone.0156261)
Supplement: S3 Table — (DOCX) [file pone.0156261.s006.docx]

|  |  | Oral cleft | | Cleft lip | | Cleft lip and palate | | Cleft palate | |
| --- | --- | --- | --- | --- | --- | --- | --- | --- | --- |
|  |  | Affected individuals, n (%) | Comparison cohort, n (%) | Affected individuals, n (%) | Comparison cohort, n (%) | Affected individuals, n (%) | Comparison cohort, n (%) | Affected individuals, n (%) | Comparison cohort, n (%) |
| Total sample | | 910 (100.0) | 9,110 (100.0) | 62 (100.0) | 620 (100.0) | 236 (100.0) | 2,360 (100.0) | 612 (100.0) | 6,130 (100.0) |
| Male | | 489 (53.7) | 4,890 (53.7) | 39 (62.9) | 390 (62.9) | 152 (64.4) | 1,520 (64.4) | 298 (48.7) | 2,980 (48.6) |
| Year of birth | |  |  |  |  |  |  |  |  |
|  | 1936−1955 | 32 (3.5) | 320 (3.5) | 5 (8.1) | 50 (8.1) | 12 (5.1) | 120 (5.1) | 15 (2.5) | 150 (2.4) |
|  | 1956−1975 | 172 (18.9) | 1,730 (19.0) | 8 (12.9) | 80 (12.9) | 52 (22.0) | 520 (22.0) | 112 (18.3) | 1,130 (18.4) |
|  | 1976−1995 | 321 (35.3) | 3,210 (35.2) | 20 (32.3) | 200 (32.3) | 85 (36.0) | 850 (36.0) | 216 (35.3) | 2,160 (35.2) |
|  | 1996−2009 | 385 (42.3) | 3,850 (42.3) | 29 (46.8) | 290 (46.8) | 87 (36.9) | 870 (36.9) | 269 (44.0) | 2,690 (43.9) |
| Number of deaths^a^ | | 90 (9.9) | 135 (1.5) | 6 (9.7) | 14 (2.3) | 38 (16.1) | 53 (2.2) | 46 (7.5) | 68 (1.1) |
| Number of suicides in 1970-2010 | | - | 10 (0.1) | - | - | - | - | - | 6 (0.1) |
| Number with any emigration^a^ | | 20 (2.2) | 392 (4.3) | - | 25 (4.0) | - | 107 (4.5) | 14 (2.3) | 260 (4.2) |
| Number of persons alive and resident in Denmark at initiation of follow up | |  |  |  |  |  |  |  |  |
|  | when earliest possible age of onset is 5 years | 828 (91.0) | 8,551 (93.9) | 54 (87.1) | 552 (89.0) | 207 (87.7) | 2,226 (94.3) | 567 (92.6) | 5,773 (94.2) |
|  | when earliest possible age of onset is 10 years | 671 (73.7) | 7,098 (77.9) | 41 (66.1) | 442 (71.3) | 173 (73.3) | 1,894 (80.3) | 457 (74.7) | 4,762 (77.7) |
|  | when earliest possible age of onset is 35 years | 180 (19.8) | 2,139 (23.5) | 12 (19.4) | 135 (21.8) | 53 (22.5) | 676 (28.6) | 115 (18.8) | 1,328 (21.7) |
| Any psychiatric disorder^a^ | | 129 (14.2) | 745 (8.2) | 7 (11.3) | 42 (6.8) | 35 (14.8) | 199 (8.4) | 87 (14.2) | 504 (8.2) |
|  | Organic, including symptomatic, mental disorder^d^ | - | 10 (0.5) | - | - | - | - | - | 6 (0.5) |
|  | Mental and behavioral disorders due to psychoactive substance abuse^c^ | 12 (1.8) | 128 (1.8) | - | 10 (2.3) | - | 32 (1.7) | 8 (1.8) | 86 (1.8) |
|  | Schizophrenia and related disorders^c^ | 7 (1.0) | 77 (1.1) | - | 7 (1.6) | - | 17 (0.9) | 6 (1.3) | 53 (1.1) |
|  | Mood disorders^c^ | 20 (3.0) | 172 (2.4) | - | 5 (1.1) | 5 (2.9) | 46 (2.4) | 13 (2.8) | 121 (2.5) |
|  | Neurotic, stress-related, and somatoform disorders^b^ | 32 (3.9) | 320 (3.7) | - | 13 (2.4) | 6 (2.9) | 79 (3.5) | 24 (4.2) | 228 (3.9) |
|  | Eating disorders | - | 38 (0.4) | - | - | - | 13 (0.6) | - | 24 (0.4) |
|  | Specific personality disorders^c^ | 9 (1.3) | 116 (1.6) | - | 6 (1.4) | - | 27 (1.4) | 6 (1.3) | 83 (1.7) |
|  | Mental retardation | 52 (5.7) | 43 (0.5) | - | - | 14 (5.9) | 8 (0.3) | 37 (6.0) | 32 (0.5) |
|  | Pervasive developmental disorders | 36 (4.0) | 84 (0.9) | - | 5 (0.8) | 10 (4.2) | 25 (1.1) | 24 (3.9) | 54 (0.9) |
|  | Behavioral and emotional disorders with onset usually occurring in childhood and adolescence | 39 (4.3) | 197 (2.2) | - | 16 (2.6) | 16 (6.8) | 61 (2.6) | 21 (3.4) | 120 (2.0) |
| Percentages is of the total sample unless otherwise stated. | |  |  |  |  |  |  |  |  |
| Number of observations less than 5 are indicated with -. | |  |  |  |  |  |  |  |  |
| ^a^ Numbers are the total number in the period April 1, 1969 to December 31, 2012. | | | | | | |  |  |  |
| ^b^ Percentages is of number of persons alive and resident in Denmark at initiation of follow up when earliest possible age of onset is 5 years of age. | | | | | | |  |  |  |
| ^c^ Percentages is of number of persons alive and resident in Denmark at initiation of follow up when earliest possible age of onset is 10 years of age. | | | | | | |  |  |  |
| ^d^ Percentages is of number of persons alive and resident in Denmark at initiation of follow up when earliest possible age of onset is 35 years of age. | | | | | | |  |  |  |
