# Supplementary material for: Psychiatric Diagnoses in Individuals with Non-Syndromic Oral Clefts: A Danish Population-Based Cohort Study
Source: PLoS One. 2016 May 25;11(5):e0156261. doi: 10.1371/journal.pone.0156261 (PMC4880322; doi:10.1371/journal.pone.0156261)
Supplement: S4 Table — (DOCX) [file pone.0156261.s007.docx]

|  |  | Individuals with oral cleft | |  | Individuals with cleft lip | |  | Individuals with cleft lip and palate | |  | Individuals with cleft palte | |
| --- | --- | --- | --- | --- | --- | --- | --- | --- | --- | --- | --- | --- |
|  |  | HR | 95% CI |  | HR | 95% CI |  | HR | 95% CI |  | HR | 95% CI |
| Any psychiatric disorder |  | 1.94*** | 1.60-2.35 |  | 1.91 | 0.86-4.25 |  | 2.01*** | 1.39-2.91 |  | 1.92*** | 1.52-2.42 |
| Organic, including symptomatic, mental disorder |  | - | - |  | - | - |  | - | - |  | - | - |
| Mental and behavioral disorders due to psychoactive substance abuse |  | 1.02 | 0.55-1.89 |  | - | - |  | - | - |  | 1.08 | 0.52-2.24 |
| Schizophrenia and related disorders |  | 0.89 | 0.39-2.04 |  | - | - |  | - | - |  | 1.26 | 0.54-2.94 |
| Mood disorders |  | 1.31 | 0.82-2.11 |  | - | - |  | 1.09 | 0.39-3.02 |  | 1.26 | 0.71-2.23 |
| Neurotic, stress-related, and somatoform disorders |  | 1.09 | 0.75-1.59 |  | - | - |  | 0.93 | 0.41-2.14 |  | 1.09 | 0.71-1.70 |
| Eating disorders |  | 0.58 | 0.14-2.42 |  | - | - |  | - | - |  | - | - |
| Specific personality disorders |  | 0.80 | 0.39-1.64 |  | - | - |  | - | - |  | 0.68 | 0.27-1.67 |
| Mental retardation |  | 13.27*** | 8.84-19.91 |  | - | - |  | 18.78*** | 7.78-45.32 |  | 12.83*** | 7.99-20.60 |
| Pervasive developmental disorders |  | 4.63*** | 3.14-6.85 |  | - | - |  | 4.54*** | 2.18-9.44 |  | 4.71*** | 2.91-7.62 |
| Behavioral and emotional disorders with onset usually occurring in childhood and adolescence |  | 2.15*** | 1.53-3.03 |  | - | - |  | 3.01*** | 1.73-5.22 |  | 1.86*** | 1.17-2.96 |
| HRs are only estimated if number of observations are 5 or more in each cohort group. | | |  |  |  |  |  |  |  |  |  |  |
| * p<0.05, **p<0.01, ***p<0.001 |  |  |  |  |  |  |  |  |  |  |  |  |
